# Folate-Targeted mRNA Delivery Using Chitosan-Functionalized Selenium Nanoparticles: Potential in Cancer Immunotherapy

**DOI:** 10.3390/ph12040164

**Published:** 2019-11-04

**Authors:** Fiona Maiyo, Moganavelli Singh

**Affiliations:** Nano-Gene and Drug Delivery Group, Discipline of Biochemistry, University of KwaZulu-Natal, Private Bag X54001, Durban 4000, South Africa; fimaiyo@gmail.com

**Keywords:** selenium nanoparticles, cancer, immunotherapy, cytotoxicity, F*luc* mRNA, transfection

## Abstract

Systemic messenger RNA (mRNA) delivery, although still in its infancy, holds immense potential for application in cancer vaccination and immunotherapy. Its advantages over DNA transfection make it attractive in applications where transient expression is desired. However, this has proved challenging due to mRNA’s instability and susceptibility to degradation. Selenium is important for immune function and modulation, with selenium nanoparticles (SeNPs) finding a niche in biomedicine as drug delivery vehicles, owing to their biocompatibility, low toxicity, and biodegradability. In this investigation, we synthesized chitosan-coated SeNPs with a folic acid targeting moiety for F*luc* mRNA delivery to cancer cells in vitro. Synthesized SeNPs were stable and well dispersed, and ranged from 59 to 102 nm in size. Nanoparticles bound and protected mRNA from RNase degradation, while exhibiting low cytotoxicity in the human embryonic kidney (HEK293), breast adenocarcinoma (MCF-7), and nasopharyngeal (KB) cells in culture. Moderate cytotoxicity evidenced in the colorectal carcinoma (Caco-2) and colon carcinoma (HT-29) cells was attributed to apoptosis induction by selenium, as confirmed by acridine orange/ethidium bromide staining. Selenium uptake studies corroborated the transfection results, where significant transgene expression was evident for the overexpressed folate receptor-positive KB cells when compared to the other cells with less or no folate receptors.

## 1. Introduction

Advancements in cancer genetics have heralded a new era of gene therapeutics, with novel approaches in gene delivery designed to permanently or transiently change phenotypes currently being investigated [1]. Gene therapy holds promise for the treatment of many genetic diseases and has primarily focused on plasmid DNA (pDNA) and small interfering RNA (siRNA). mRNA-based gene therapy has several advantages over pDNA. Primarily, mRNA does not need to enter the nucleus to function, a challenge faced by DNA delivery, and is hence a safer alternative, with a reduced risk of insertional mutagenesis [2,3]. Unlike DNA where the strength of the promoter determines expression, mRNA is easier to engineer and does not require incorporation of a promoter and a terminator construct, enhancing its attractiveness as a gene therapeutic [4,5,6]. Nanoparticle-mediated mRNA transfection favours the combination of different therapeutic mRNAs on one carrier, allowing for adjustment by changing the amount and type of mRNA transfected. Despite these advantages over DNA, delivery of mRNA as a gene therapeutic has only recently re-emerged as it was previously deemed too unstable to work with. Stability has been increased through several modification strategies to improve the feasibility of mRNA for in vitro and in vivo studies [7,8]. 

Evasion of the immune system, a hallmark of cancer, occurs through the secretion of immune-suppressive cytokines, which downregulates major histocompatibility complex (MHC) molecules [9]. The tumour-associated antigens on the tumour cell surface trigger recognition by the host’s immune system. Another strategy for cancer immunotherapy lies in utilizing genetically modified immune cells, viz. T and dendritic cells to respond to tumour antigens [10]. The numerous mutations in the coding exons of cancer cells are potential targets for antigens, with studies reporting the delivery of mRNA encoding tumour-associated antigens to dendritic and tumor cells [11,12]. Cancer vaccination offers a new form of cancer treatment with the potential to improve therapeutic outcomes by triggering the patient’s immune system to eliminate the tumour. Intratumoural therapeutic mRNA delivery is still in its infancy, with preliminary investigations in melanoma patients using a polycation protamine, showing increased immune response against cancer [13].

Safe and efficient delivery of a nucleic acid to a target site of action remains a major obstacle to successful gene therapy, halting many clinical trials [13]. The success of therapeutic mRNA delivery has been further hindered by mRNA instability, inefficient delivery and uptake, and limited transfection [10]. Core–shell nanoparticles composed of inorganic and organic materials are efficient delivery vehicles due to their defined physical features, tunable size, and versatile chemical and physical properties, which can be used over a wide range of gene and drug delivery platforms [14,15,16,17], in addition to their multifunctional capabilities, enhanced biocompatibility, and synergistic properties. To date, there are very few reports on their use for mRNA delivery and almost none on an inorganic/organic core shell system.

Selenium nanoparticles (SeNPs) have found a niche in drug delivery, owing to their biocompatibility, bioactivity, and biodegradability in vivo. Selenium (Se), a potent antioxidant, is an essential trace element, important for the function of many enzymes in the body [15,18], with its deficiency affecting glutathione metabolism and protein synthesis by reducing circulating cysteine and homocysteine, a risk factor for cardiovascular diseases, viral infections, and cancer [19]. Se also plays an important role in immune function, with deficiencies resulting in a weakened defence against disease, as well as a reduction in the ability to metabolise drugs [20]. Se has been reported to reverse tumor-mediated immunosuppression, with preclinical and clinical reports confirming their immune modulation effects [21,22,23].

Chitosan, a positively charged natural polysaccharide made up of β (1-4)-glucosamine and N-acetyl-D-glucosamine is one of the most attractive polymers for gene delivery, owing to its cationic nature, low cytotoxicity, low immunogenicity, and biocompatibility. It has been successfully used in the delivery of pDNA and siRNA [24,25,26,27]. Herein, we evaluate the transfection efficiency of F*luc*-mRNA conjugated to chitosan-coated SeNPs, a biodegradable core–shell carrier with a folate targeting moiety, as a potential nanodelivery vehicle to cancer cells in vitro. This study aimed at formulating a therapeutic mRNA nanocarrier with a synergistic effect for potential cancer immunotherapy.

## 2. Results and Discussion

### 2.1. Preparation and Characterization of Nanoparticles

**Figure 1:**
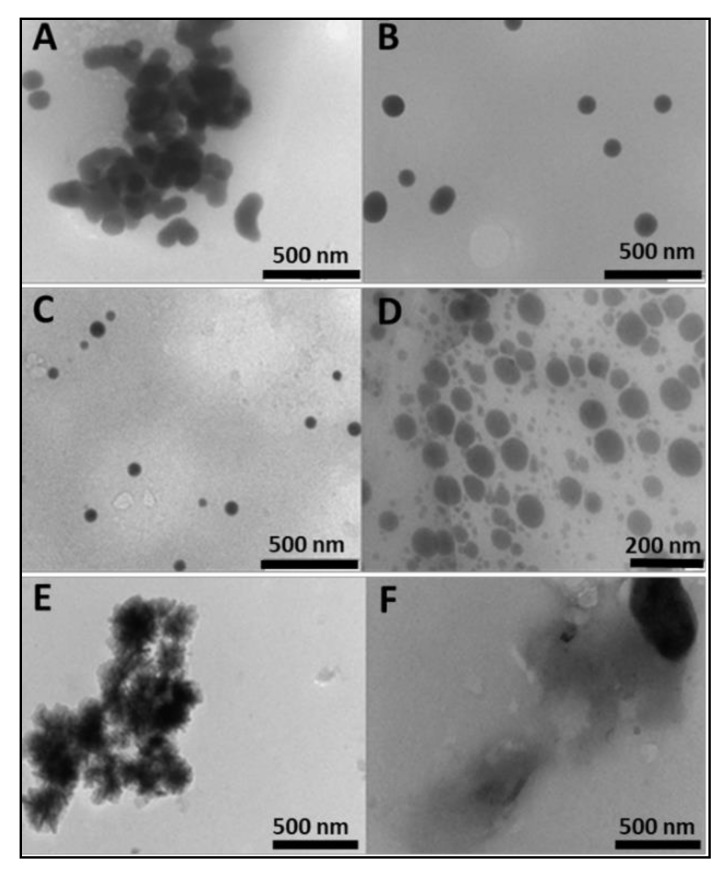
Transmission electron microscopy (TEM) images of (**A**) and (**B**) SeNPs, (**C**) SeCh, (**D**) SeChFA, (**E**) Ch, and (**F**) ChFA.

Sodium selenite was reduced to red colloidal selenium with ascorbic acid and functionalised with chitosan in a one-pot synthesis technique. The hydroxyl groups of chitosan reacted with SeO_3_^2−^, which was then reduced to SeNPs by ascorbic acid [28]. Previous studies have demonstrated successful chitosan encapsulation of SeNPs, which increased cellular retention of the nanoparticles [29,30]. The SeNPs were predominantly spherical in shape, as seen under transmission electron microscopy (TEM) (Figure 1), with an average size of 85.3 ± 8 nm and a negative zeta (ζ) potential (−14.8 ± −3.6 mV) (Table 1). The encapsulation of SeNPs with chitosan immediately brought about a reduction in size (59.6 nm) and a reversion to a positive zeta potential (21.0 ± 0.2 mV), resulting in monodisperse particles and an increase in colloidal stability. Increased homogeneity has been reported when 0.5% chitosan was used in functionalisation [31], as adopted in this study. These results confirmed that chitosan functionalization was key in modulating size, charge, and dispersity. Conjugation of the targeting moiety, folic acid (FA) to chitosan through carbodiimide chemistry, was confirmed by spectroscopic studies. The degree of FA substitution as determined by UV spectroscopy was 1.9%, which we attribute to the high molecular weight of chitosan, which has been reported to decrease FA substitution [32]. Degrees of substitution of 0.58–2.2% have been used successfully in gene and drug delivery [27,33,34]. The particle size of SeChFA increased (75.6 ± 1.4 nm), with a corresponding reduction in ζ potential (9.0 ± 0.3 mV), which could be attributed to substitution of amino groups with FA, as well as the possible shielding of the positive charges by FA. However, a positive surface charge was maintained, which was essential for mRNA binding and association with the anionic cell membrane. Stability of all NPs was monitored after storage at 4 °C for two months, with no significant differences in size and ζ potential observed. The polydispersity values for all nanoparticles and nanocomplexes were low and ranged from 0 for SeCh NPs to 0.00475 for the SeChFA NPs, while the nanocomplexes ranged from 0.0001 for ChFA–F*Luc* mRNA to 0.0025 for the SeCh–F*Luc* mRNA nanocomplexes (Table 1). This suggests that all nanoparticles and nanocomplexes were monodisperse. It was reported that PDI values between 0 and 1 indicate an ideal monodisperse system [35]. Overall, except for the non-functionalized SeNPs, all other NPs and nanocomplexes displayed moderately high ζ potentials, indicating good colloidal stability, as very low or high zeta potential can result in aggregation in vivo. The respective NTA profiles for the nanoparticles and nanocomplexes are presented in the Supplementary Material (Figures S1–S3).

**Figure 2:**
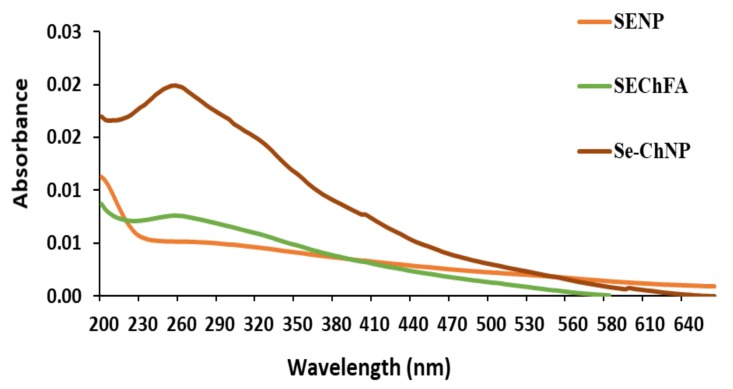
UV characterizations of synthesized and functionalized selenium nanoparticles.

UV–Vis studies further confirmed successful synthesis and functionalisation of SeNPs (Figure 2). The non-functionalised SeNPs had an absorbance maximum at a wavelength < 200 nm and SeCh at 254 nm, with a slight shift upon FA conjugation to 257 nm, indicating successful conjugation. A single absorbance peak revealed the presence of spherical particles. 

FTIR studies revealed the presence of the characteristic functional groups of chitosan and FA on functionalised SeNPs, confirming conjugation of FA to chitosan via the amide linkage. The vibration at 3200 cm^−1^ widened due to the overlap of −OH and N−H groups. COO− stretching at 1603 cm^−1^ on FA disappeared upon chitosan conjugation. Prominent 1670 cm^−1^ and 1584 cm^−1^ vibrations assigned to amide I and amide II confirmed the amide bond formation and chitosan attachment (Supplementary Figure S4–S5).

### 2.2. mRNA Binding and Nuclease Protection Studies

**Figure 3:**
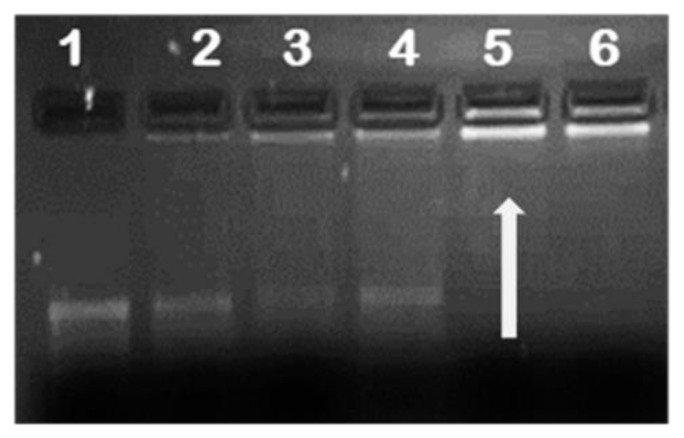
Representative image of mRNA/nanoparticle (NP) binding. Binding studies of SeCh to F*luc* mRNA. Lane 1 contained naked mRNA and served as control. Lanes 2–6 contained nanocomplexes of mRNA (0.2 µg) and NPs at different w/w ratios. Arrow indicates end-point ratios of mRNA/NP (w/w).

It is essential for any gene delivery vehicle to successfully compact and protect the nucleic acid from the extracellular matrix during transport to its cellular target. To investigate the mRNA loading capacity of the NPs, complexes at different NP/mRNA (w/w) ratios were prepared and subjected to agarose gel electrophoresis to establish the optimum binding ratio. Results showed successful binding of all functionalised NPs to mRNA with FA-containing NPs having the higher endpoint ratios (Table 1). This was probably due to the reduced positive charge as a result of the substitution of the amino group with FA masking some of the positive charges. Figure 3 provides a representative image of mRNA binding.

**Figure 4:**
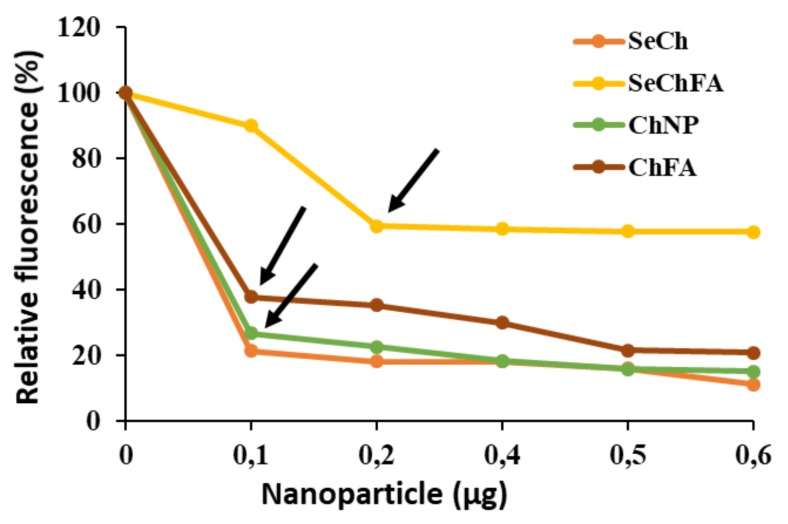
Ethidium Bromide displacement. Arrows indicate end-points.

The high fluorescence upon ethidium bromide intercalation into mRNA was successfully quenched by the addition of the cationic NPs, as the dye was displaced from the nucleic acid (Figure 4). These results correlated with end-point ratios obtained from gel retardation, showing SeCh and Ch NPs binding to mRNA at a lower concentration than the targeted ChFA and SeChFA. 

**Figure 5:**
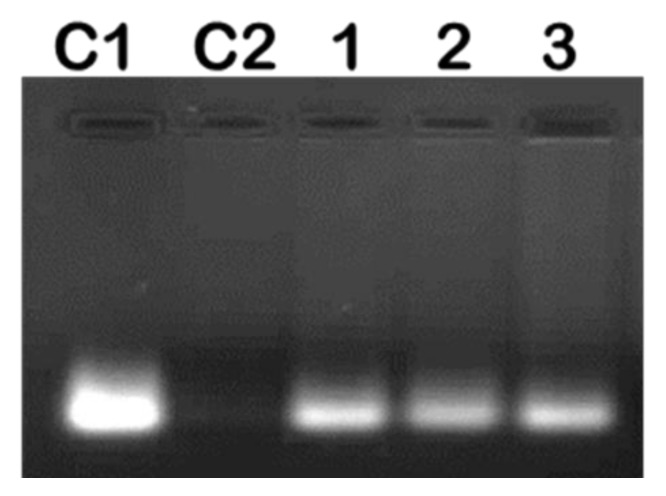
Representative image showing RNase A digestion of mRNA/SeChFA nanocomplexes. C1 = F*luc* mRNA (0.2 µg) only and C2 F*luc* mRNA exposed to RNase A. Lanes 1–3 represent nanocomplexes at suboptimum, optimum, and supraoptimum binding ratios.

Complexing of mRNA with the NP should ensure protection from nuclease degradation, which was investigated at the suboptimum, optimum, and supraoptimum ratios. After incubation with RNase A for 2 h, naked mRNA was completely degraded, with very little or no degradation for the nanocomplexes observed (Figure 5). This confirmed that these NPs afforded significant protection to their mRNA cargo.

### 2.3. Cytotoxicity Studies

**Figure 6:**
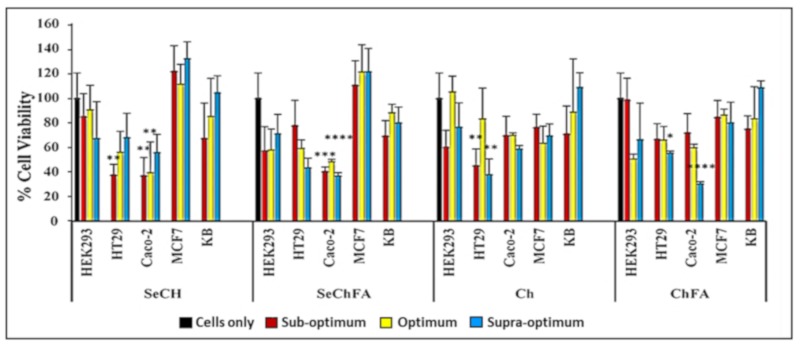
Cell viability studies of nanocomplexes at suboptimum, optimum, and supraoptimum ratios in selected cell lines. Data are presented as mean ± SD (n = 3). ^****^ *p* < 0.0001; ^***^ *p* < 0.001; ^**^ *p* < 0.01; ^*^ *p* < 0.05 vs. control.

The MTT assay was used to evaluate the effect of the nanocomplexes on cell viability in vitro (Figure 6). SeChFA and SeCh exhibited some cytotoxicity in the Caco-2 cell line across all binding ratios with cell viabilities below 50%. This could be due to the increased Se required for mRNA binding and the presence of FA increasing cellular uptake in the folate receptor-positive Caco-2 cell line, as reported previously [34]. The non-targeted NPs showed low cytotoxicity across all cell lines within the concentration used in transfection. High transfection efficiency is the ultimate goal, and the degree of cytotoxicity plays a key role in its success. Apoptosis induction compromises transfection pathways and could be the main cause of the toxicity observed in Caco-2 cells.

Moderate toxicity was observed on the second colon cancer cell line HT29 for the targeted nanocomplexes. Chitosan and ChFA nanocomplexes were well tolerated across all cell lines; however, there was significant toxicity in the Caco-2 cells with the ChFA nanocomplexes at the highest ratio. Chitosan is a reported antioxidant that quenches the ROS formation of selenium, reducing its cytotoxicity. Selenium nanocomplexes were well tolerated in the MCF-7 and nasopharyngeal (KB) cells, with lower toxicity in the non-cancer control HEK293 cells (Figure 6). Overall, good cell viability was observed, with mild toxicity in some cell lines, which can be attributed to selenium’s apoptosis-inducing activity, as evidenced by the acridine range/ethidium bromide apoptosis study. The pathogenesis and genotype of a cancer cell varies from one tumour to another; thus, their sensitivity to compounds will differ, as evidenced in this study.

### 2.4. In Vitro Luciferase Expression

**Figure 7:**
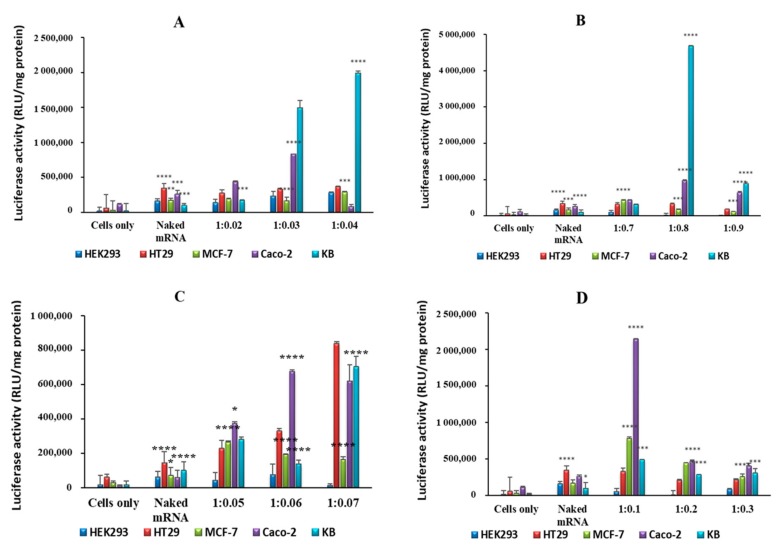
Luciferase activity in selected cell lines using (**A**) SeCh, (**B**) SeChFA, (**C**) CH, and (**D**) ChFA nanocomplexes at different NP/mRNA w/w ratios. Data are presented as means ± SD (n = 3). ^****^ *p* < 0.0001; ^***^ *p* < 0.001; ^**^ *p* < 0.01; ^*^ *p* < 0.05; vs. control.

Transfection of exogenous mRNA allows for transient expression of proteins, which are either absent, altered, or in low concentrations. In vitro gene expression was evaluated using the luciferase reporter gene assay in receptor-negative and -positive cell lines, with HEK293 being a non-cancer, receptor-negative control cell line. Higher levels of gene expression were evident for all nanocomplexes compared to naked mRNA in all cell lines. Transfection with selenium-containing nanocomplexes showed much higher transfection than simple chitosan nanocomplexes, especially in the folate receptor-rich KB cell line. Despite a low positive ζ potential, SeChFA nanocomplexes showed enhanced transfection at the optimum binding ratio, which was also significantly higher than the ChFA nanocomplexes (Figure 7). It has been reported that a low ζ potential is preferable in some cases of targeted delivery, since it can hinder non-specific binding and uptake into non-targeted cells [36]. Furthermore, chitosan encapsulation of selenium has been reported to increase cellular uptake, while reducing the pro-oxidative activity of selenium that may lead to DNA damage [37], and has been demonstrated to successfully deliver pDNA, siRNA, and chemotherapeutic drugs. SeCh NPs were also reported to impede mRNA translation of proteins responsible for oncogenesis and cell proliferation [38].

**Figure 8:**
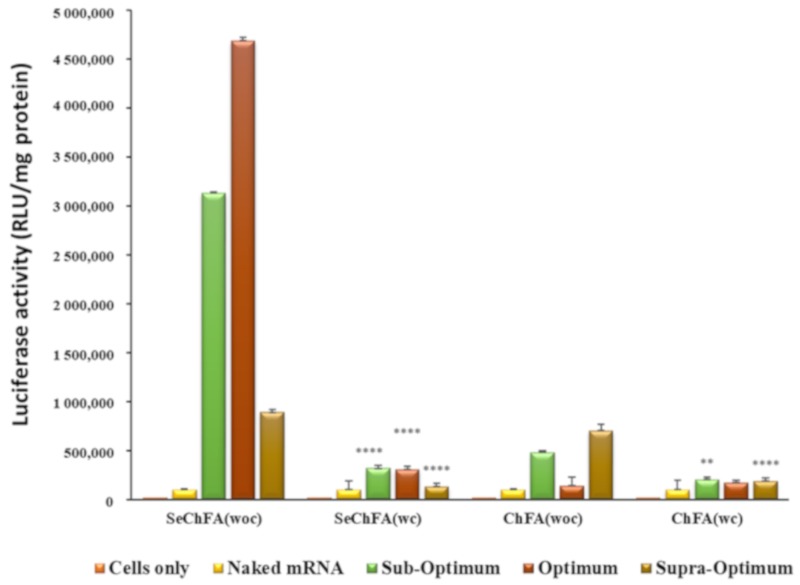
Competition assay of targeted nanocomplexes in nasopharyngeal (KB) cells showing luciferase expression with FA competitor (wc) and without the FA competitor (woc). Data are presented as means ± SD (n = 3). ^****^ *p* < 0.0001; ^***^ *p* < 0.001; ^**^ *p* < 0.01; ^*^ *p* < 0.05; vs. luciferase activity without competitor.

Since the receptor-rich KB cells produced significantly high transgene expression, a competition assay was conducted to confirm the entry of the nanocomplexes by receptor mediation. Lower luciferase activity was recorded for cells treated with excess free FA prior to transfection, signifying that folate receptor targeting was the most probable pathway of entry into these cells. The addition of free FA thus reduced cellular uptake through receptor-mediated endocytosis by competitively binding to the receptor. The ChFA nanocomplexes were not severely affected, with only a slight decrease in transfection after addition of the competitor (Figure 8). However, the SeChFA nanocomplexes showed a significant drop in gene expression. 

### 2.5. Selenium Uptake

**Figure 9:**
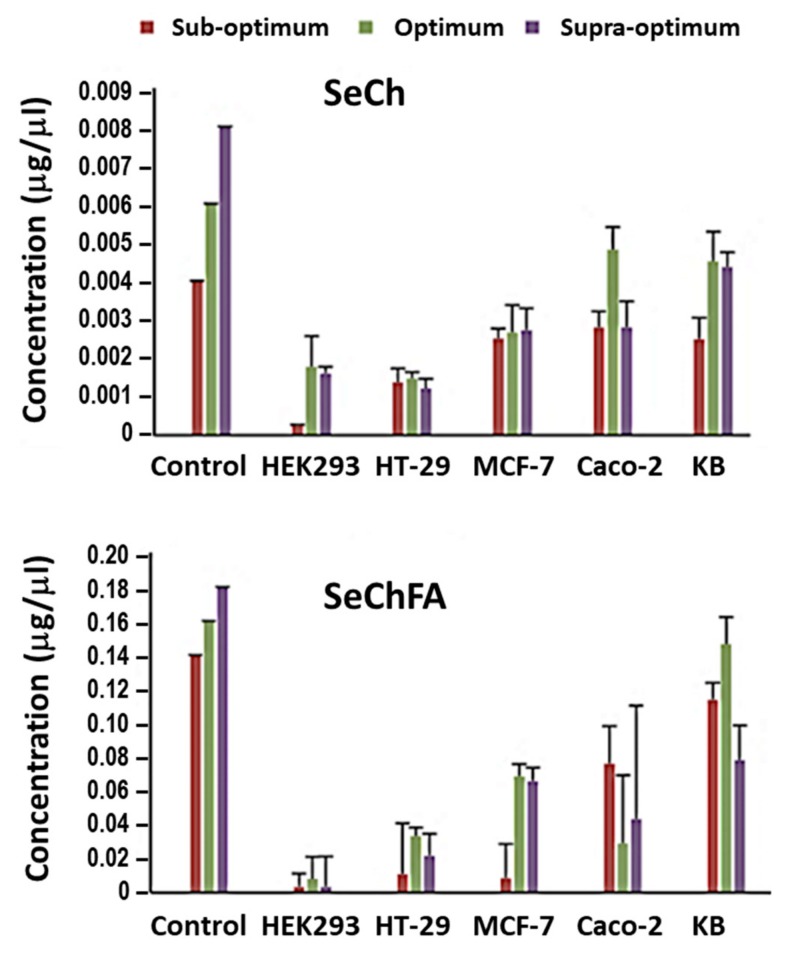
Selenium concentration in the cells before (control) and after transfection with SeCh and SeChFA.

Cellular uptake of Se was quantified using inductively coupled plasma-optical emission spectroscopy (ICP-OES) 24 h after transfection. Higher concentrations of Se were recorded after transfection with the targeted SeChFA than with SeCh NPs. Considering that a lower concentration of Se was used in nanocomplex formation of the latter, this was expected and correlated with the transfection results. Furthermore, data obtained (Figure 9) showed that not all of the Se added before transfection was internalised, which was in agreement with that reported in previous literature [39], which demonstrated uptake and localisation of chitosan functionalised SeNPs in HepG2 cells.

### 2.6. AO/EB Dual Staining

**Figure 10:**
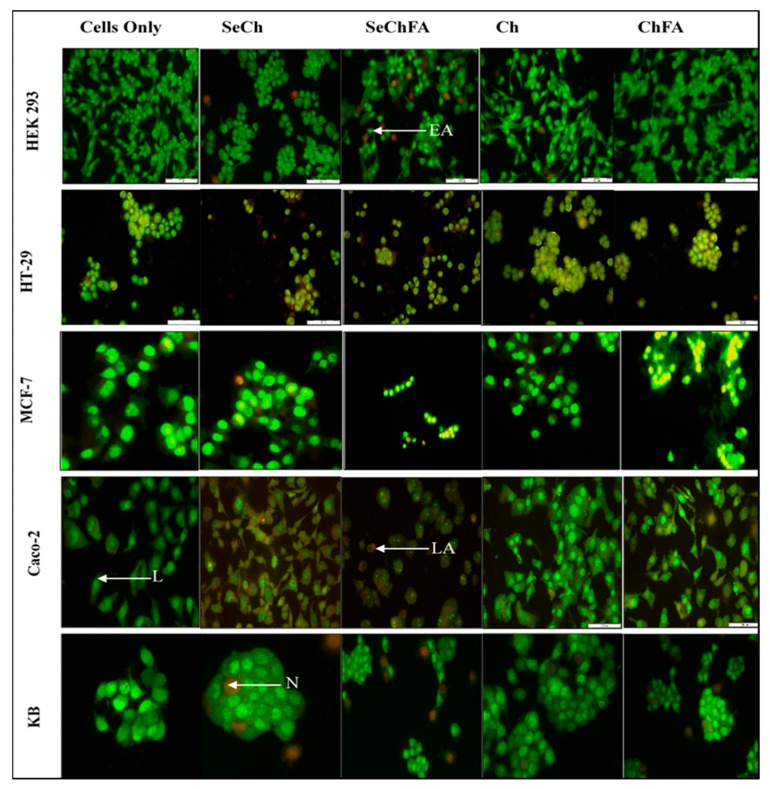
Apoptosis studies of nanocomplexes at optimum binding ratios on HEK293, HT-29, MCF-7, Caco-2, and KB cell lines. L = live cells, EA = early apoptotic, LA = late apoptotic, N = necrotic.

We investigated apoptosis induction as a possible mechanism of toxicity based on the MTT assay results. Acridine Orange/Ethidium Bromide (AO/EB) staining enabled the study of apoptosis through microscopic examination of changes in the cell membrane and the nucleus of cells, clearly distinguishing normal cells and those in different stages of apoptosis. Acridine orange is taken up by both viable and early apoptotic cells, while ethidium bromide is only taken up by the non-viable necrotic cells whose membrane integrity has been compromised, making their nucleus to fluoresce bright orange [39]. Apoptotic cells appear bright green to yellow with a granular or crescent-shaped nucleus. Late apoptotic cells display an asymmetrical nucleus coloured bright yellow and orange. A granular yellow/green crescent-shaped nucleus characterizes early apoptotic cells, while late stage apoptotic cells have an asymmetrical nucleus and necrotic cells increased in volume with a large rounded orange nucleus (Figure 10). Apoptosis was observed in cells transfected with Se nanocomplexes, but at very low apoptotic indices (AI), except in Caco-2 cells where the indices ranged from 0.71 to 1 (Table 2). Selenium compounds are known to induce apoptosis at certain concentrations. The concentration used for transfection in this study was low and based on ICP studies where less than 50% was taken up by the cells. Targeting of selenium increased uptake in receptor-positive cell lines, which would explain the higher indices for SeChFA in Caco-2 cells. However, this was not seen for the KB cells, despite similar uptake to Caco-2, with necrosis being the primary form of cell death.

## 3. Materials and Methods 

### 3.1. Materials

Sodium selenite, ascorbic acid, chitosan (>75% deacetylated; MW 218 kDa), MTT (3-(4,5-dimethyldiazol-2-yl)-2,5-diphenyltetrazolium bromide), folic acid, N, N’-dicyclohexyl-carbodiimide (DCCI), RNase, and bicinchoninic acid (BCA) solution were all purchased from Sigma–Aldrich (St. Louis, MO, USA). Agarose, 2-[4-(2-Hydroxyethyl)-1-piperazinyl] ethane sulphonic acid (HEPES), tris(hydroxymethyl)-aminomethane hydrochloride (Tris-HCL), EDTA, SDS (sodium dodecyl sulphate) ethidium bromide, and acridine orange were purchased from Merck (Darmstadt, Germany). Luciferase assay reagent kit and reporter lysis buffer were supplied by Promega (Madison, USA). Eagle’s Minimum Essential Medium (EMEM) with Glutamax^TM^, penicillin/streptomycin (10,000 U mL^−1^ penicillin, 10,000 U mL^−1^ streptomycin) and trypsin-versene were supplied by Lonza Biowhittaker (Walkersville, USA). All sterile plasticware were obtained from Corning Inc. (NY, USA). Cell lines were purchased originally from the American Type Culture Collection (Manassas, VA, USA). Fetal bovine serum (FBS) was obtained from Hyclone, GE Healthcare (Utah, USA). Lyophilised F*luc* mRNA modified with 5-methylcitidine and pseudouridine was purchased from TriLink BioTechnologies Inc (San Diego, CA, USA). This was dissolved in nuclease-free water to a concentration of 0.05 µg mL^−1^. All other chemicals used in this study were of analytical grade, and Milli-Q water (18 MΩ) was used throughout.

### 3.2. Preparation and Modification of Selenium Nanoparticles (SeNPs)

SeNPs were prepared as previously described [31], with slight modifications. Briefly, 5 mM sodium selenite (8.7 mg, 10 mL) was added dropwise to 20 mM ascorbic acid (35.2 mg, 10 mL) and adjusted to a final concentration of 1 mM. The mixture was stirred at room temperature for 24 h and stored at 4 °C. Selenium chitosan nanoparticles (SeCh NPs) were synthesized with modifications [29]. Approximately 10 mL of 0.5% chitosan (in 1% acetic acid) was added to 7.5 mL of 0.23 M ascorbic acid and 5 mL of 18 Mohm water. The mixture was stirred under low heat for 30 min, followed by addition of 0.25 mL of 0.51 M sodium selenite, causing a colour change from colourless to red. After stirring for 2 h at room temperature, the nanoparticles (NPs) were dialysed (MWCO 12 kDa) against 18 MΩ water over 24 h.

Folic acid (FA)-targeted SeCh NPs (SeChFA NPs) were prepared as per literature [32], but modified using carbodiimide chemistry for FA conjugation. Approximately 40 mg of FA and 100 mg of DCCI were dissolved in 15 mL of anhydrous DMSO and stirred for 1 h at room temperature. Thereafter, 20 mL of chitosan was added dropwise to the activated FA with stirring for 24 h. The pH was adjusted to 9.0, and the coagulated mixture centrifuged at 2500 rpm for 10 min. The supernatant of the ChFA conjugate was dialysed (MWCO 12 kDa) against 18 MΩ water over 48 h. SeChFA NPs were prepared by the addition of ChFA dropwise to the prepared SeNPs with stirring for 12 h. The content of FA in the ChFA conjugate was analysed using UV-Vis spectroscopy at 359 nm, and the coupling ratio was calculated using the formula: Coupling Ratio = W(FA)/W(FA-Ch)—W(FA)

### 3.3. mRNA/NP Binding 

The mRNA nanocomplexes were prepared by adding varying amounts of functionalized SeNPs to 0.2 µg of F*luc* mRNA, followed by a 30 min incubation at room temperature to allow for the formation of the nanocomplexes through electrostatic interactions. Nanocomplexes were then subjected to 2% agarose gel electrophoresis (50 V, 30 min) containing ethidium bromide (1 µg/mL) to determine optimal binding ratios, as assessed by the electrophoretic mobility shift of the mRNA bands. The fluorescent bands were viewed, and images captured using a Vacutec Syngene G: Box Imaging system (Syngene, Cambridge, UK). The optimal binding ratio, one ratio above (supraoptimal) and one ration below (suboptimal), was used in further studies.

### 3.4. Ethidium Bromide Intercalation Assay

Ethidium bromide (EB) dye displacement was used to study the degree of compactness of the nanocomplexes based on the quenching of fluorescence upon the addition of the functionalized SeNPs to an EB/mRNA mixture. Approximately 2 µL of EB (100 µg/µL) was added to 100 µL HBS in a black multi-well plate and fluorescence measured in a Glomax® Multidetection system (Promega Biosystems, Sunnyvale, USA) at an excitation and emission wavelength of 520 nm and 600 nm, respectively. This was set as the baseline fluorescence reading. Approximately 4.8 µL mRNA was then added to the mixture, and the reading set as 100% fluorescence. Thereafter, 1 µL aliquots of the respective NPs were added and mixed, and individual readings obtained, until a plateau in fluorescence was achieved. 

### 3.5. RNase A Protection Assay

To investigate the stability of nanocomplexes and the protection afforded to the mRNA by the functionalized SeNPs if exposed to serum nucleases, an RNase A protection assay was carried out. Nanocomplexes at optimal, sub-, and supraoptimal ratios obtained from Section 2.3 were incubated with 1 µL of RNase A at 37 °C for 2 h. Thereafter, EDTA (10 mM) and SDS (0.5% w/v) for nanocomplex dissociation were added, and mixtures were incubated at 55 °C for 20 minutes. Nanocomplexes were subjected to agarose gel electrophoresis as previously described (Section 2.3). Naked mRNA and mRNA treated with RNase A were used as positive and negative controls, respectively. 

### 3.6. Nanoparticle and Nanocomplex Characterization

UV-Vis spectroscopy was conducted using a JASCO-V-730 BIO spectrophotometer in the range of 190–500 nm. FTIR was carried out to further characterize the nanoparticles. The IR spectra for functional group identification were obtained on a Perkin–Elmer Spectrum 100 FTIR spectrometer with a universal ATR sampling accessory scanning from 4,000 to 380 cm^−1^. The shape, size, and distribution of NPs and nanocomplexes were analysed by TEM (JEOL JEM 1010), operating at 100 kV. The NP/nanocomplex suspension (10 µL) was placed on copper grids and allowed to dry at room temperature. Images were analysed using iTEM Soft Imaging Systems (Tokyo, Japan). Particle size, distribution, and zeta potential measurements were obtained from nanoparticle tracking analysis (NTA) (NanoSight NS500; Malvern Instruments, UK) operating at 25 °C and 24 V, using NTA version 3.2 software. Samples (1 mL in 18 MΩ water) were prepared at approximate concentrations of 10^8^ particles/mL. 

### 3.7. Cell Viability Assays 

Cells (HEK293, MCF-7, KB, Caco-2, and HT-29) were seeded at a density of 1.8 × 10^5^ cells per well in 48-well plates, containing 200 µL of medium (EMEM + Glutmax supplemented with 10% FBS and antibiotics (100 U/mL penicillin, 100 µg/mL streptomycin)), and allowed to attach overnight at 37 °C. Thereafter, medium was replenished with 200 µL fresh medium, and complexes at the three ratios were added in triplicate. Cells were incubated for 48 h at 37 °C. The medium was then replaced with 200 µL medium containing 20% MTT (5 mg/mL in PBS), and cells were incubated for 4 h at 37 °C. Thereafter, the medium/MTT mixture was removed, and 200 µL of DMSO was added to each well to dissolve the resulting formazan product. Absorbance was read in a Mindray 96A microplate reader (Vacutec, Hamburg, Germany) at 570 nm. All tests were conducted in triplicate.

### 3.8. Gene Expression

The F*luc* mRNA reporter gene encoding the firefly luciferase gene was used to investigate the transfection efficiencies in the five cell lines. Cells were seeded at a density of 4.5 × 10^4^ per well in a 48-well plate, containing complete medium, and incubated overnight for attachment. Thereafter, fresh medium was added to the cells, and nanocomplexes (as previously described) were added to the cells in triplicate. Untreated cells and cells treated with naked F*luc* mRNA (0.2 µg) served as controls. The cells containing nanocomplexes were incubated for 48 h at 37 °C. Thereafter, the medium was discarded, and cells were washed twice with PBS. Approximately 80 µL of cell lysis buffer was then added to the cells, and the plate gently shaken for 15 minutes. The cells were then scraped from the surface of the plate, and the cell lysates centrifuged at 12,000 g for 5 s to pellet the cell debris. Approximately 20 µL of the respective cell-free lysates were transferred to a white multiwell plate, followed by the addition of 100 µL of luciferase assay reagent. Luminescence was read on a Glomax® Multidetection system (Promega BioSystems, Sunnyvale, USA). Total protein was determined using a standard BCA assay (562 nm), and results were presented as relative light units (RLUs) per mg protein.

For the receptor competition assay, cells were seeded as above. However, 30 minutes prior to addition of the nanocomplexes, 50 mM of free FA was added to cells. Following a 48 h incubation, the luciferase assay was conducted as described.

### 3.9. Selenium Uptake

Detection and quantification of the levels of selenium post transfection were measured using ICP-OES, performed on a Perkin Elmer Optima 5300 DV Optical Emission Spectrometer. Transfection was done as described earlier, and after 48 h, the cells were washed with PBS and lysed with cell lysis buffer. Cell lysates were transferred into ICP vials for analysis. A standard calibration curve was set up between 0.2 ppm and 25 ppm using a standard stock solution.

### 3.10. Acridine Orange/Ethidium Bromide Dual Staining

AO/EB staining was performed to study cell apoptosis post transfection as described previously [40,41]. Cells at a density of 1.2 × 10^5^ cells/well were plated into a 24-well plate and incubated overnight for attachment. Thereafter, complexes were added to the cells at their various binding ratios and incubated for 24 h. The cells were then washed twice with PBS, and 10 μL of AO/EB dye (100 μg/mL AO and 100 μg/mL EB in PBS) was added. Cells were stained at room temperature for 5 min. Thereafter, the dye was removed, and the cells were viewed under an Olympus inverted fluorescence microscope fitted with a CC12 fluorescent camera (excitation filter of 450–490 nm and a barrier filter of 520 nm) (Wirsam Scientific and Precision Eq. LTD., Johannesburg, South Africa) at X200 magnification. Cells were examined for morphological changes due to apoptosis. Apoptosis was represented as an index, calculated as shown below:

*Apoptotic index = number of apoptotic cells/number of total cells counted* (1)

### 3.11. Statistical Analysis

Experiments were carried out in triplicate, and data were presented as means ± SD. Statistical analyses were done using two-way ANOVA on GraphPad Prism Version 5.04 (GraphPad Software Inc. USA), followed by Bonferroni post hoc tests, which analysed the differences between the means. *P*-value < 0.05 was considered significant.

## 4. Conclusions

A chitosan-coated selenium vector in mRNA delivery has the potential for use in tumour vaccination and immunotherapy to achieve an amplified immune response. Our goal was to develop a functionalised Se carrier capable of mRNA delivery to tumour cells as a proof of concept study for use in cancer immunotherapy, and is the first report of its use in mRNA delivery. Functionalized SeNPS were able to safely and stably deliver the mRNA cargo in vitro, with the inclusion of the FA-targeting moiety further increasing uptake in folate receptor-positive cells. The use of chitosan-coated selenium NPs in mRNA delivery has the potential for application in tumour vaccination and immunotherapy. This study has shown that there may exist a future synergy between RNA vaccines and SeNPs, which bodes well for immunotherapy. Compelling evidence from this research makes further studies necessary to fully understand mRNA interaction with selenium for gene therapy applications.

## Figures and Tables

**Table 1:** Size, zeta potential, and polydispersity (PDI) of all nanoparticles and nanocomplexes at optimum binding w/w ratios.

NP	Size (nm)	ζ Potential (mV)	PDI	Nanocomplexes at end-Point Ratios (Optimum Binding Ratio)			
				w/w	Size	ζ Potential	PDI
**Se**	85.3 ± 8	−14.8 ± −3.6	0.00450	-	-	-	
**SeCH**	59.6 ± 0.1	21.0 ± 0.2	0	1:0.03	66.9 ± 0.9	14.4 ± 1.7	0.0025
**SeChFA**	75.6 ± 1.4	9.0 ± 0.3	0.00475	1:0.8	102.7 ± 15.2	9.8 ± 0.3	0.0006
**CH**	124.4 ± 19	38.5 ± 2.7	0.00064	1:0.06	162.9 ± 5.9	40.7 ± 1.3	0.0012
**ChFA**	139.5 ± 3.5	21.9 ± 0.7	0.00370	1:0.2	136.1 ± 18.5	32.9 ± 1.5	0.0009

**Table 2:** Apoptotic indices post transfection with nanocomplexes at optimum binding.

Cell Lines	Apoptotic Index				
	Control	SeCh	SeChFA	Chitosan	ChFA
HEK293	0	0	0.11	0	0
HT29	0	0.15	0.09	0.11	0.23
MCF7	0	0.07	0.28	0	0
Caco-2	0	0.71	1	0.02	0.83
KB	0	0.04	0	0	0.02

## Supplementary Materials

The following are available online at https://www.mdpi.com/1424-8247/12/4/164/s1, Figure S1: NTA profiles of (A)SeNPs, (B) SeCh NFPs and (C) SeChFA NPs. Figure S2: NTA profiles of (A) SeCh and (B) SeChFA: F*Luc*-mRNA nanocomplexes. Figure S3: NTA profiles of (A) Ch, (B) ChFA, (C) Ch: F*Luc*-mRNA and (D) ChFA: F*Luc*-mRNA. Figure S4: FTIR of (A) Chitosan, (B) Folic acid and (C) Chitosan-Folic acid. Figure S5: FTIR of (A) SeCh NPs and (B) SeChFA NPs.
