# Supplementary material for: Folate-Targeted mRNA Delivery Using Chitosan-Functionalized Selenium Nanoparticles: Potential in Cancer Immunotherapy
Source: Pharmaceuticals (Basel). 2019 Nov 4;12(4):164. doi: 10.3390/ph12040164 (PMC6958489; doi:10.3390/ph12040164)
Supplement: Supplementary file 1 [file pharmaceuticals-12-00164-s001.pdf]

Supplementary Material

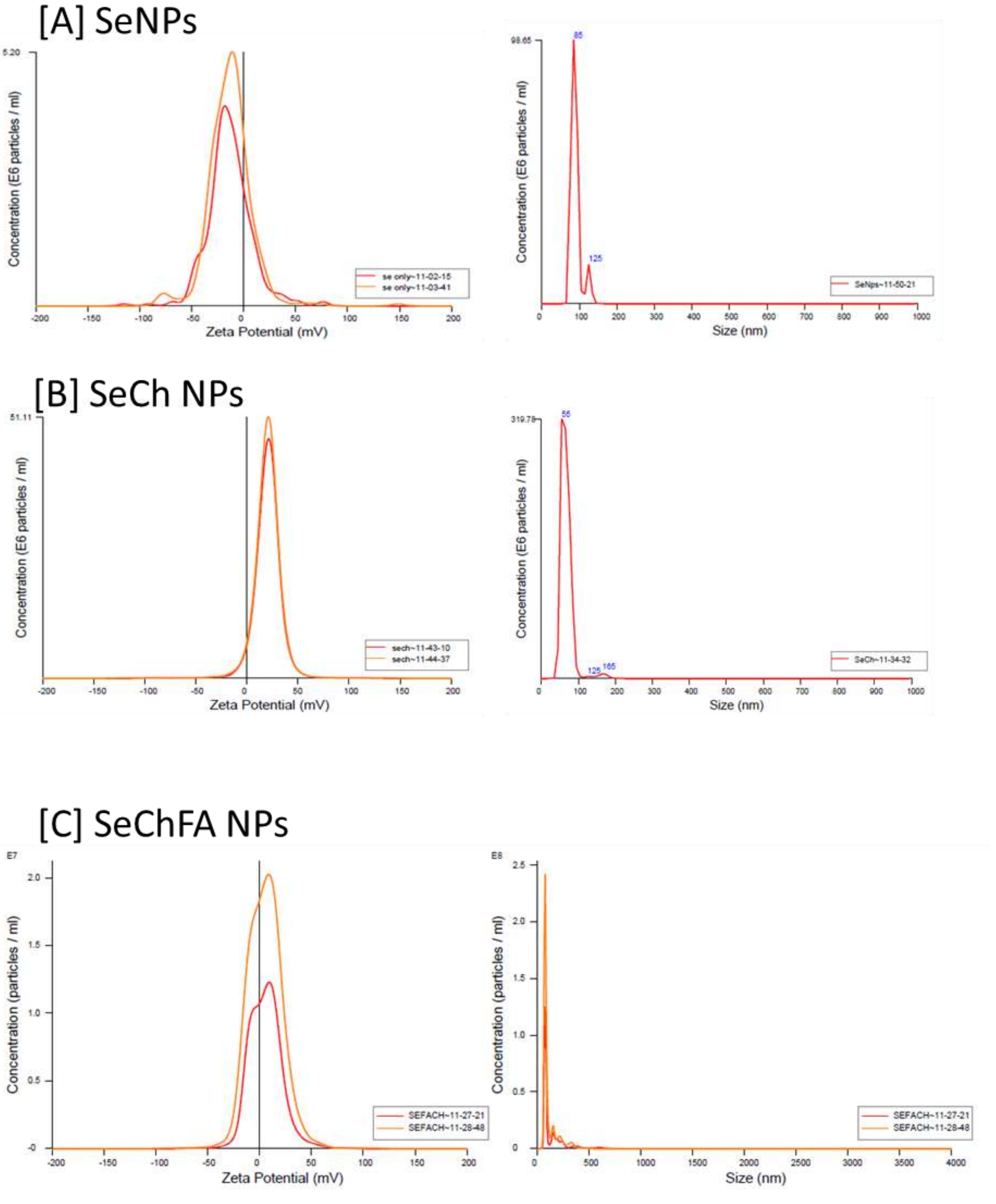

Figure S1. NTA profiles of (A)SeNPs, (B) SeCh NPs and (C) SeChFA NPs.

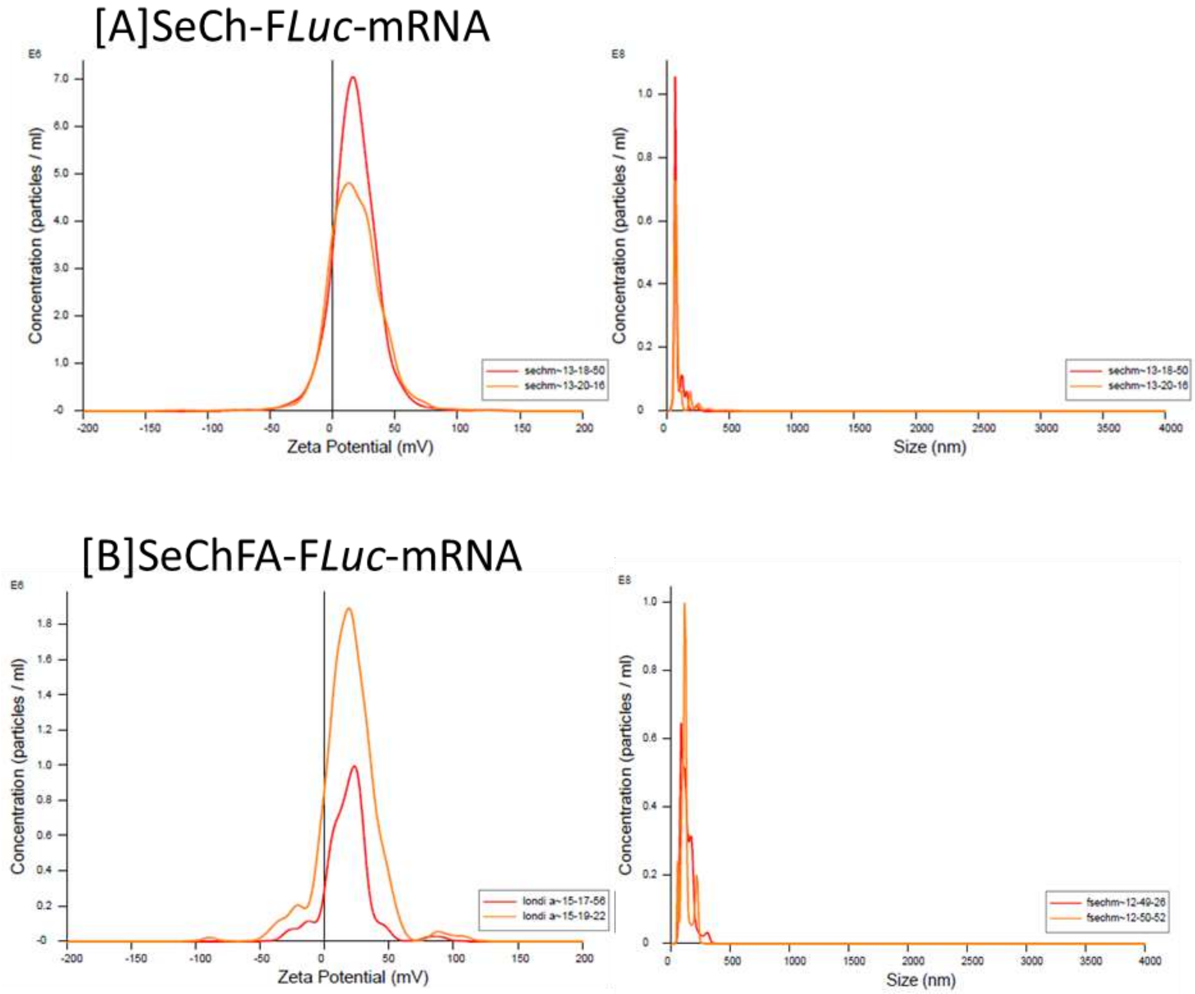

Figure S2. NTA profiles of (A) SeCh and (B) SeChFA: F*Luc*-mRNA nanocomplexes.

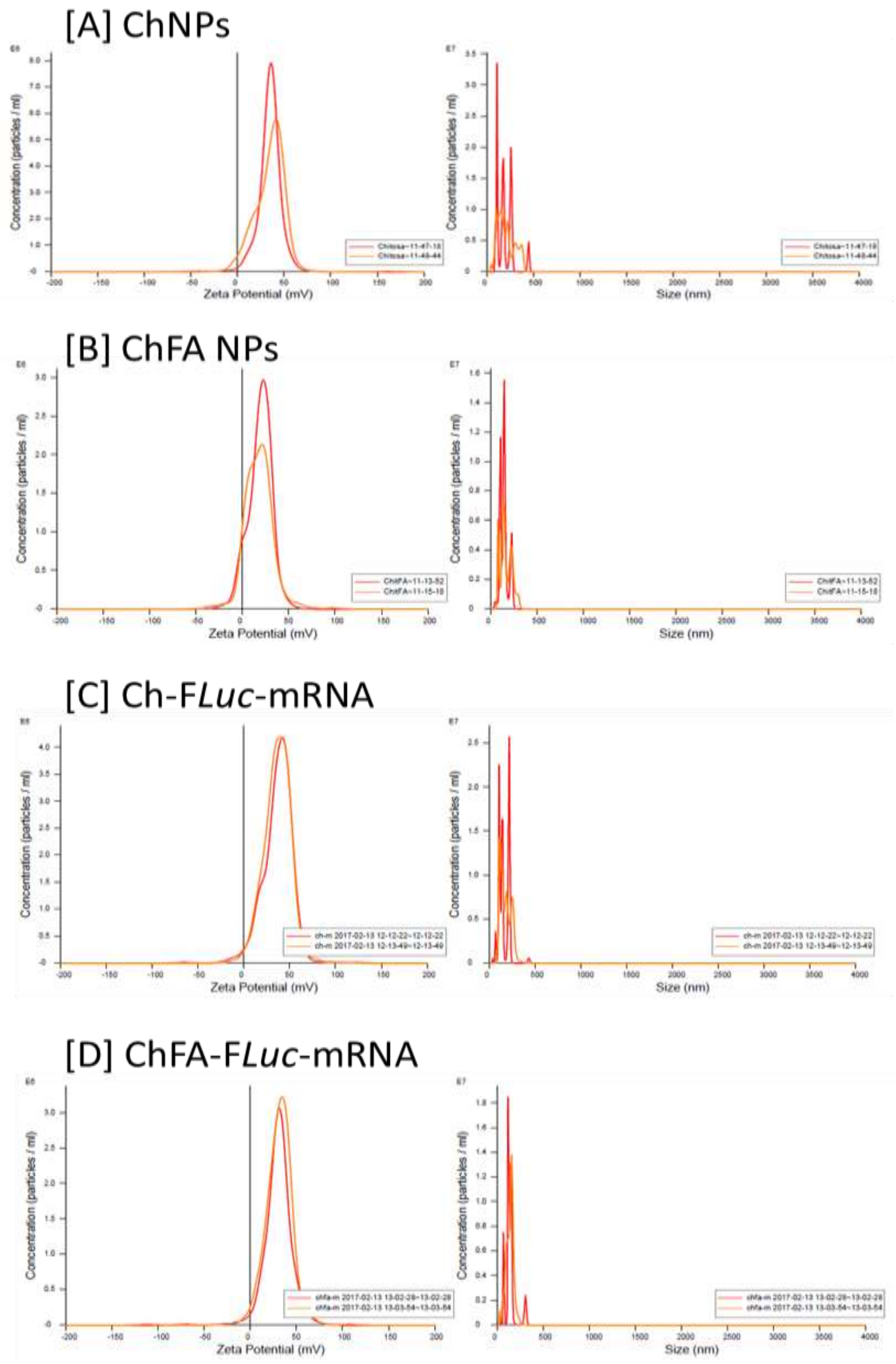

Figure S3. NTA profiles of (A) Ch, (B) ChFA, (C) Ch: F*Luc*-mRNA and (D) ChFA: F*Luc*-mRNA.

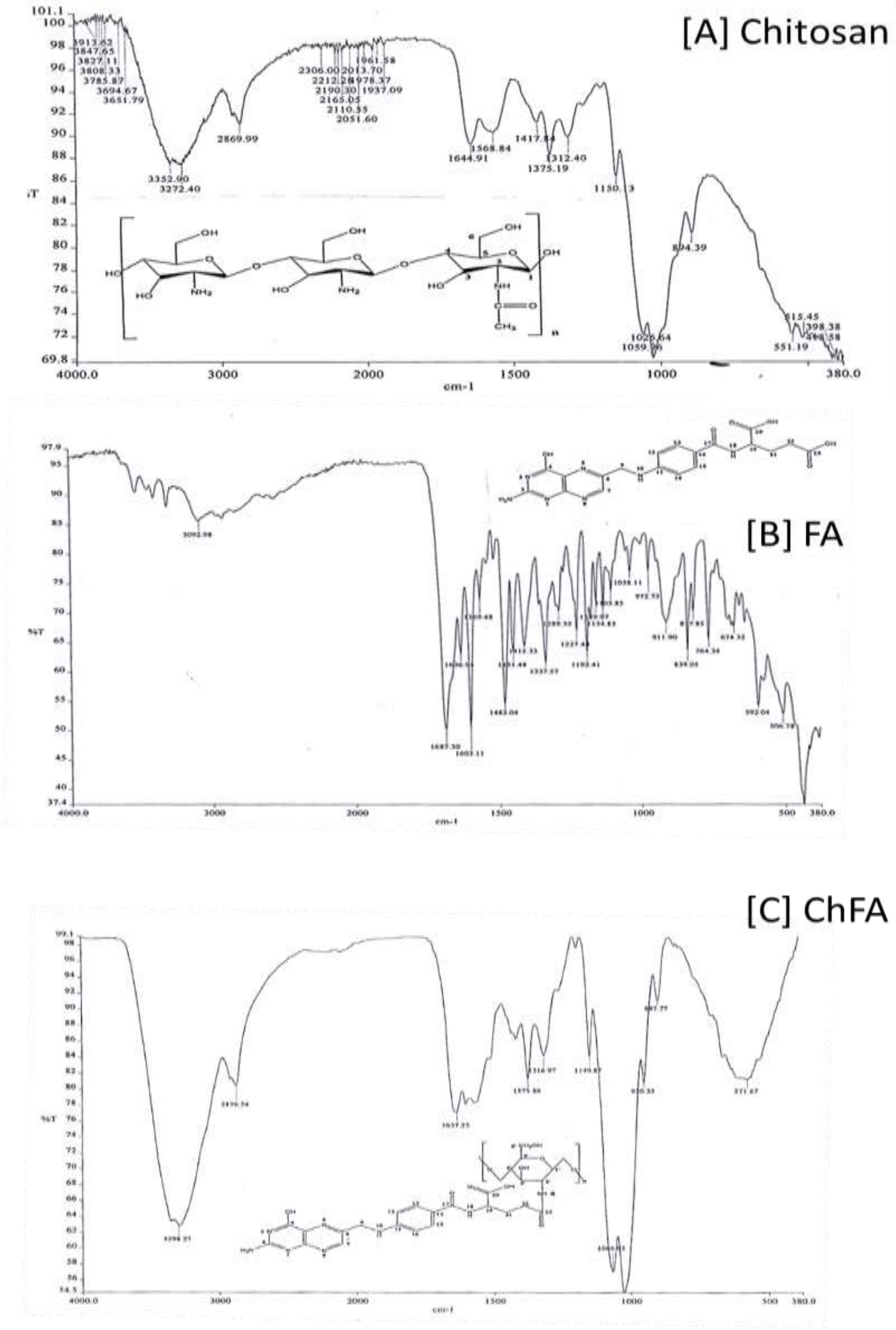

Figure S4: FTIR of (A) Chitosan, (B) Folic acid and (C) Chitosan-Folic acid.

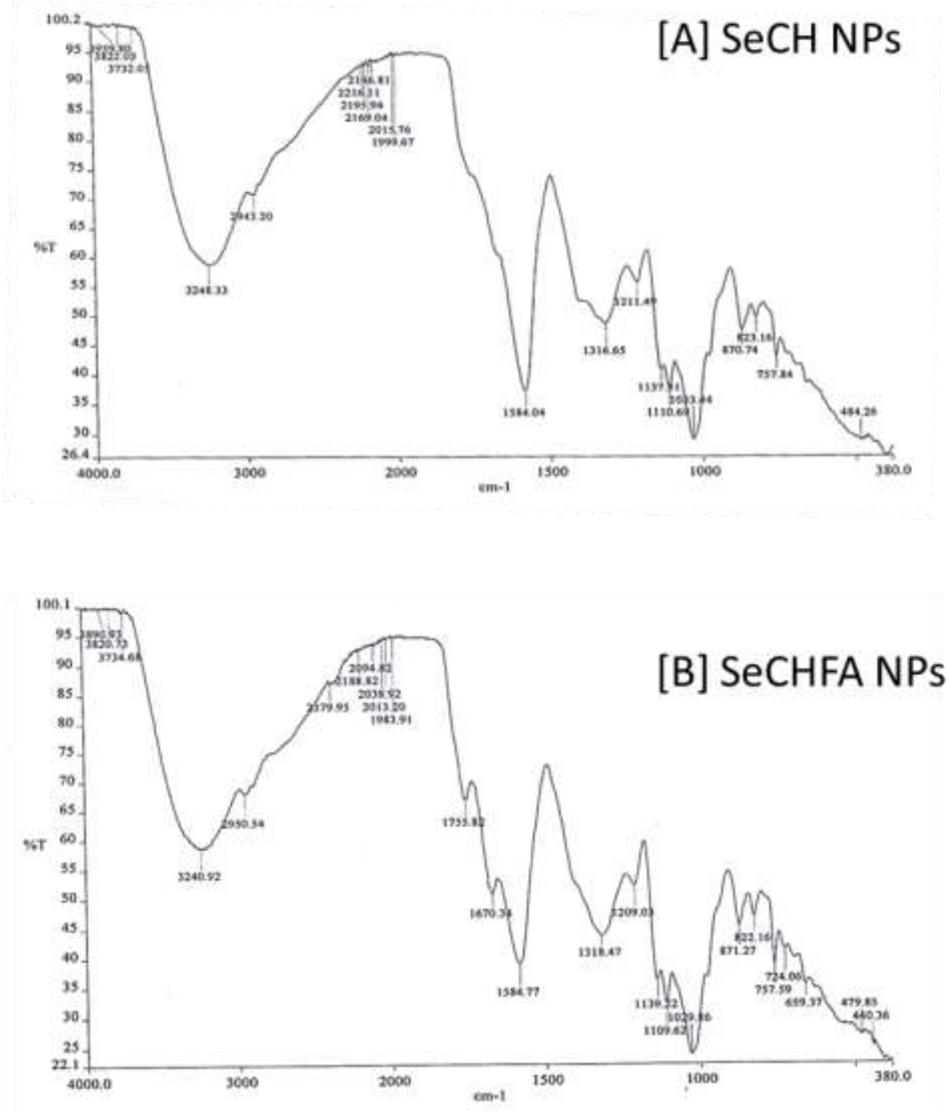

Figure S5: FTIR of (A) SeCh NPs and (B) SeChFA NPs.
